# Sox5 and Th17 cell differentiation

**DOI:** 10.18632/oncotarget.4784

**Published:** 2015-07-03

**Authors:** Akira Suto, Shigeru Tanaka, Hiroshi Nakajima

**Affiliations:** Department of Allergy and Clinical Immunology, Graduate School of Medicine, Chiba University, Chiba, Japan

**Keywords:** immunology and microbiology section, immune response, immunity

Sox (SRY-related high-mobility-group (HMG)-box) transcription factors, which are comprised of 20 genes containing a conserved HMG DNA-binding domain, are divided into 8 groups (from A to H) according to structural homologies [reviewed in 1–2]. SoxC group, which is composed of Sox4, Sox11, and Sox12, has a HMG box domain and a transactivation domain and plays important roles in the development of heart, nerve system, kidney, and pancreas. SoxD group, which is composed of Sox5, Sox6, and Sox13, has a HMG box domain in C-terminal half and group-specific coiled-coil domain(s) in N-terminal half. SoxD proteins interact with each other through their coiled-coil domain and their activity is likely to be influenced by other molecules with which they interact. Sox5 and Sox6 are paralogous genes highly expressed in spermatids, neurons, oligodendrocytes, and chondrocytes, and are indispensable for chondrogenesis.

Regarding the roles of Sox family genes in T cell immunity, it has been demonstrated that Sox4 suppresses GATA3 function and thereby inhibits Th2 cell-mediated inflammation [reviewed in 3]. On the other hand, Sox13 plays a critical role in the development of γδ T cell receptor_+_ thymocytes and Sox13, together with Sox4, induces the differentiation of IL-17-producing γδ T cells (Tγδ17 cells) through the induction of orphan nuclear receptor RORγt [3]. Sox4 is also expressed in αβ T cells; however, Sox4 is not essential for the differentiation of IL-17-producing αβ T cells (Th17 cells) [3], which are involved not only in the host defense against extracellular pathogens but also in the pathogenesis of autoimmune diseases [4]. Because RORγt, which is encoded by *Rorc* gene, plays a central role in the differentiation of Th17 cells as well as Tγδ17 cells [4], it is suggested that the involvement of Sox transcription factors in RORγt induction seems different between Th17 cells and Tγδ17 cells.

**Figure 1:**
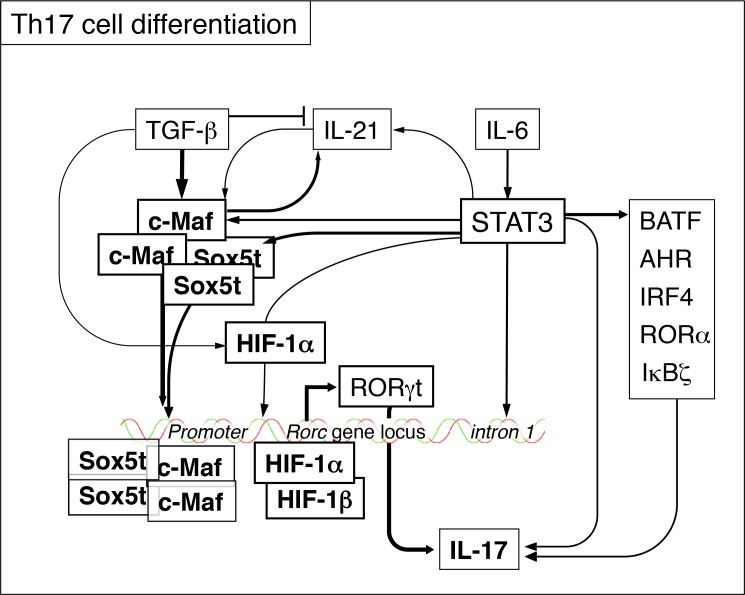
A schematic model for the role of Sox5t and c-Maf in Th17 cell differentiation

It is well established that IL-6- and/or IL-21-mediated Stat3 activation is indispensable for the induction of RORγt during Th17 cell differentiation. Stat3 binds to intron 1 of Rorc gene and induces chromatin remodeling of the locus. In addition, Stat3 activation results in the induction of many genes implicated in Th17 cell differentiation such as Nfkbiz, Rora, Batf, Irf4, Ahr, Maf, and HIF-1α (Figure 1) [4]. Among these genes, HIF-1 has been shown to activate Rorc promoter. However, the downstream targets of Stat3 for RORγt induction in Th17 cells have not been fully understood. To identify the downstream pathway of IL-6-Stat3 signaling for Th17 cell differentiation, we have performed DNA microarray analysis of IL-6-stimulated CD4_+_ T cells. We found that c-Maf and a novel isoform of Sox5 (named Sox5t) were highly induced in IL-6-stimulated CD4_+_ T cells and that Stat3 was indispensable for the induction of c-Maf and Sox5t [5]. We also found that severity of Th17 cell-mediated immune responses, such as experimental autoimmune encephalomyelitis and delayed-type hypersensitivity, were reduced in T cell-specific Sox5-deficient mice [5]. Moreover, Th17 cell differentiation was impaired in T cell-specific Sox5-deficient mice both in vivo and in vitro, indicating that Sox5t is involved in Th17 cell differentiation.

To address the mechanism underlying Sox5t-mediated Th17 cell differentiation, we examined the effect of retrovirus-mediated Sox5t induction on Th17 cell differentiation. Intriguingly, although the enforced expression of Sox5t itself did not induce IL-17 production in CD4_+_ T cells, the expression of Sox5t together with c-Maf significantly induced IL-17 production even under neutral conditions [5]. Co-induction of Sox5t and c-Maf induced IL-17 production in Stat3-deficient CD4_+_ T cells but not in RORγt-deficient CD4_+_ T cells, suggesting that Sox5t and c-Maf induce Th17 cell differentiation as downstream effectors of Stat3 and as upstream inducers of RORγt. We further demonstrated that RORγt was one of the direct targets of Sox5t and c-Maf by integrating the data of RNA-seq analyses and ChIP-seq analyses of Sox5t- and c-Maf-expressed CD4_+_ T cells. Moreover, we confirmed that Sox5t and c-Maf activated RORγt promoter in primary CD4_+_ T cells. Analyses using deletion mutants of Sox5t and c-Maf revealed that Sox5t was associated with c-Maf via HMG domain of Sox5t and DNA-binding domain of c-Maf.

Our study has demonstrated that Sox5t is expressed in Th17 cells and together with c-Maf, induces RORγt expression during Th17 cell differentiation (Figure 1). With regard to the relationship between Sox5 and autoimmune diseases in humans, it has been shown that Sox5 is one of the most strikingly upregulated transcription factors in whole blood in patients with multiple sclerosis [6]. In addition, a genome-wide association study (GWAS) has revealed that Sox5 is associated with limited systemic sclerosis [7]. Taken together, these results suggest the involvement of Sox5 expressed in CD4_+_ T cells in the pathogenesis of autoimmune diseases through the induction of Th17 cell differentiation.
